# Undergraduate nursing students’ self-efficacy in clinical teaching: a cross-sectional study

**DOI:** 10.1186/s12912-025-04165-8

**Published:** 2025-11-27

**Authors:** Beth Pierce, Jeanne Allen, Thea van de Mortel

**Affiliations:** Griffith University, Brisbane, Australia

**Keywords:** Clinical teaching, Nurse, Pioneering study, Self-efficacy, Student, Undergraduate

## Abstract

**Background:**

Effective clinical teaching by nurses supports the transfer of knowledge, skills and values amongst nursing students, nurses and health professionals, contributing to safe quality healthcare. Worldwide, professional nursing bodies include clinical teaching as a requirement for nursing registration. Despite this, graduating nursing students judge their clinical teaching capabilities to be less developed than other competencies. To enhance nursing students’ clinical teaching capabilities, research suggests they should first strengthen their self-efficacy related to clinical teaching; however, little, if anything, is known about the development of nursing students’ clinical teaching self-efficacy. This study aimed to examine the development of undergraduate nursing students’ self-efficacy in clinical teaching during their degree program.

**Methods:**

A cross-sectional survey design was used. Year 2 and final year (Year 3) nursing students in an Australian Bachelor of Nursing program completed the Modified Self-Efficacy in Clinical Teaching Scale. Multiple linear regression identified the predictors of clinical teaching self-efficacy and *t*-tests determined year-level differences. Item response frequencies and means were used to identify the clinical teaching tasks with which participants were most and least confident.

**Results:**

For the total sample (*n* = 319), participants’ year level of study (*p* = .002) and peer teaching experience (*p* < .001) were significant positive predictors of clinical teaching self-efficacy. Year 3 participants’ clinical teaching self-efficacy was higher than that of Year 2 participants (*p* < .001). Year 2 and 3 participants were most confident they could teach with prowess and least confident they could impact a learner’s development. More than half of Year 3 students rated their confidence with clinical teaching tasks as moderate or low and Year 2 participants were less confident across all clinical teaching tasks compared to Year 3 participants.

**Conclusions:**

This study examined nursing students’ clinical teaching self-efficacy – an underreported topic. Results suggest that nursing students *can* develop their clinical teaching self-efficacy throughout their degree program. To enhance nursing students’ clinical teaching self-efficacy, university educators should consider integrating peer teaching experiences and best practice clinical teaching principles into curricula. This research supports future benchmarking of nursing students’ self-efficacy outcomes with those of other disciplines and degree programs.

## Background

Nurses have a role as educators, with a professional responsibility to teach patients, groups, students, other nurses, and health professionals in a variety of healthcare contexts [1]. In nursing, the education of students, other nurses, and health professionals in clinical settings is termed *clinical teaching* [2]. Effective clinical teaching has been found to support the transfer of knowledge, skills and professional values among students, nurses and health professionals, ultimately contributing to healthcare safety and quality [2, 3]. 

Since 1987, the International Council of Nurses [4] has included the education of nursing students, other nurses and health professionals within their longstanding definition of the role of a nurse. Professional nursing bodies, such as the Nursing and Midwifery Board of Australia [5], the Nursing Council of New Zealand [6] and the United Kingdom’s Nursing and Midwifery Council [7] include clinical teaching as a requirement for professional nursing registration. Clinical teaching is also an important part of nurse professional competence. In developing their Holistic Nursing Competence Scale, Takase and Teraoka [8] emphasised the importance of nurses educating, guiding and managing other nurses and staff. They deemed the creation of learning and teaching environments and cultures to be an essential part of the nursing role. Nilsson and colleagues [9] similarly identified clinical teaching as a vital component of nursing professional competence. They included the education and supervision of staff and students as one of the eight areas of nursing practice that underpin professional competence [9]. 

While clinical teaching is an integral component of professional nursing standards and competence, research suggests that nurses and nursing students have little or no formal education related to clinical teaching or adult learning theory [10, 11]. Accordingly, some graduating nursing students judge their clinical teaching capabilities (i.e., their capabilities to teach other nursing students, nurses and/or health professionals) to be less developed than other nursing competencies. In 2019, Nilsson and colleagues [12] surveyed more than 700 undergraduate nursing students across six European countries. They reported that graduating students perceived their ability to educate and supervise other students and staff upon graduation as the lowest of their professional competencies. In Australia, van de Mortel and colleagues [13] similarly found that graduating nursing students rated their teaching and supervision skills as low compared to other nursing skills. This is concerning, given that some newly graduated nurses are expected to supervise and educate nursing students in their first six months of professional practice [14]. 

According to Bourne and colleagues [2], to develop clinical teaching capabilities and confidence, nurses should be supported to enhance their self-efficacy beliefs related to clinical teaching. Self-efficacy beliefs, a central construct in Bandura’s Social Cognitive Theory [15], refers to a person’s belief about their abilities to perform at levels that can positively influence their lives. Bandura [15] suggested that self-efficacy relates to one’s confidence with a task or domain (i.e., clinical teaching in this study), thus differentiating self-efficacy beliefs from personality traits like self-concept or self-esteem. A person’s self-efficacy beliefs related to a particular task are thought to influence their motivation, effort and participation with that task [15]. Therefore, when a person has strong self-efficacy beliefs related to a task, they are more likely to persevere longer and achieve success with that task, even when it becomes difficult [15]. 

A person’s self-efficacy beliefs do not strengthen over time as a matter of course. A study by Liu and colleagues [16] found that almost 90% of undergraduate students in various disciplines experience little to no change in their self-efficacy beliefs during their university studies. Bandura [15] contended that to enhance a person’s self-efficacy beliefs, one must engage with four self-efficacy sources. One of these sources, *vicarious experiences*, involves a person observing someone else successfully complete a task, leading them to believe they too have the potential to succeed with that task [15]. A second source is *verbal persuasion*, whereby a person is told by someone else that they are competent to complete a task and able to succeed [15]. A third self-efficacy source is *physiological and affective states*, which involve a person perceiving a task to be joyful or exciting, eliciting little stress or anxiety [15]. A fourth self-efficacy source, *mastery experiences*, involves a person’s repeated engagement with meaningful, challenging and effortful tasks. Of the four self-efficacy sources, mastery experiences are thought to have the greatest impact on a person’s self-efficacy beliefs [15]. 

In the field of education, much has been written about self-efficacy beliefs related to teaching. In 2015, Chesnut and Burley [17] conducted a metanalysis of 33 studies that reported on the self-efficacy beliefs of more than 16,000 pre-service and qualified teachers from North America, Europe, Asia and Australia. They found a positive relationship between pre-service and qualified teachers’ self-efficacy in teaching and their teaching commitment, which was deemed a reflection of their motivation to teach. Educational researchers Klassen and Tze [18] also conducted a metanalysis involving 43 studies and 9,200 teachers. They determined that teachers’ self-efficacy beliefs had a statistically significant effect on their teaching effectiveness and enthusiasm, as rated by their students or supervisors.

In the medical and nursing literature, health professionals’ self-efficacy in teaching has been associated with both teaching willingness and teaching performance. Hayat and colleagues [3] determined that doctors with strong clinical teaching self-efficacy beliefs were more likely to be prepared for clinical teaching and to demonstrate best practice principles of clinical teaching. In nursing, the teaching self-efficacy beliefs of nurses were found to contribute to their readiness to engage with health teaching, specifically in the areas of asthma education and postpartum care [1, 19, 20]. 

University undergraduate degree programs are essential for preparing nursing students for clinical teaching upon graduation [21]. Despite this, little, if anything, is known about the extent to which undergraduate nursing students develop their self-efficacy in clinical teaching throughout their degree program. This study addresses a gap in the literature by examining the development of undergraduate nursing students’ clinical teaching self-efficacy, an under studied topic. It presents the results of a cross-sectional clinical teaching self-efficacy survey administered to Australian undergraduate nursing students, which examined students’ beliefs in their abilities to teach more junior nursing students.

## Aims

This study aimed to examine the development of undergraduate nursing students’ self-efficacy in clinical teaching during their degree program. The research questions were:

1. What factors predict undergraduate nursing students’ self-efficacy in clinical teaching?

2. Are final year (Year 3) undergraduate nursing students more self-efficacious in clinical teaching compared to Year 2 undergraduate nursing students?

3. With which aspects of clinical teaching are undergraduate nursing students most and least confident?

## Methods

### Study design and theoretical framework

This study used a cross-sectional survey design. The research was underpinned by Bandura’s Social Cognitive Theory [15] and the theoretical construct of self-efficacy. Aligned with this theory and construct, the authors defined nursing students’ clinical teaching self-efficacy as their beliefs in their abilities to teach more junior nursing students. We assumed that these beliefs could be impacted (strengthened or diminished) through engagement, or lack thereof, with one or more self-efficacy sources.

### Instrument

A modified version of McArthur’s [22] Self-efficacy in Clinical Teaching (M-SECT) instrument and a coversheet of demographic questions was administered to participants. The M-SECT instrument consisted of 21 clinical teaching items/tasks, grouped into three domains. *Domain 1: Customising Teaching to a Learner’s Needs* examined a person’s confidence with appraising the needs of a learner, providing appropriate instructional content and selecting teaching strategies that suit a learner’s needs [22]. *Domain 2: Teaching Prowess* centred on a person’s confidence with demonstrating clinical skills, evaluating a learner’s performance, providing feedback and showing concern for a learner’s wellbeing [22]. *Domain 3: Impact on a Learner’s Development* focused on a person’s confidence with planning and setting educational objectives that would impact a learner’s attitudes and continue to engage them, even if they are disinterested in the content [22]. 

The original Self-efficacy in Clinical Teaching instrument was administered to Australian general practitioners in clinical education roles [22] and to Australian osteopathy educators [23]. It demonstrated excellent reliability with a Cronbach’s alpha value of 0.95 [22]. The M-SECT instrument was also validated with undergraduate nursing students [24]. The instrument demonstrated excellent reliability (Cronbach’s alpha of 0.98) and its face validity, construct validity and criterion-related validity were confirmed [24]. 

### Participants and context

For this study, a sample size of approximately 285 participants was sought to achieve a 95% confidence level with a 5% margin of error [25]. Convenience sampling was used to recruit Year 2 and final year (Year 3) nursing students enrolled in a three-year (full-time equivalent) Bachelor of Nursing program at an urban university in South-east Queensland, Australia. The Bachelor of Nursing program included a constructivist nursing curriculum, which integrated increasingly complex concepts/skills and included clinical placement experiences (work-integrated learning) across year levels. As is the case with most Australian educational institutions, the program was delivered in accordance with the calendar year. Students commenced their year-level coursework in February and finished in October (the same year), with some clinical placements occurring in November and December as required.

At the time of recruitment, Year 1 students had *not* participated in a clinical placement experience and had not been exposed, theoretically or practically, to clinical teaching; thus, they were excluded from the study. Year 2 students had participated in approximately 240 h of clinical placement experiences (including residential aged care and acute settings) and had foundational knowledge of teaching and supervising in the context of intra- and interprofessional care. Year 3 students had participated in approximately 560 h of clinical placement experiences in acute, mental health, community and complex care settings. At the time of recruitment, they had learnt about the concepts of clinical teaching in the context of professional nursing responsibilities. They had also learnt about and practiced patient education in classrooms, clinical laboratories and clinical placement settings.

### Data collection

In August and September 2022 (towards the end of the year-level coursework period), prospective participants were approached by one of the researchers (who was unknown to the cohort of students) during their on-campus classes. Students were provided with a verbal explanation of the study and a Project Research Information Sheet, which detailed the study aims, procedure, risks and benefits. The Project Research Information Sheet included a statement explaining that survey completion indicated consent. The anonymous paper-based survey took approximately 10 min to complete and no identifiable participant information was collected. To reduce potential bias and coercion, a secure box was available for the return of both complete and incomplete surveys (i.e., for those participants not wishing to complete the survey).

### Data analysis

Survey data were entered into Lime Survey [26] and imported into the Statistical Package for the Social Sciences version 28.0 [27]. Before data analysis, item frequencies and minimum and maximum values were calculated to determine missing responses and out-of-range values [28]. Out-of-range values were checked with the paper surveys and corrected if needed. Because the analysis required total scores from the Modified Self-efficacy in Clinical Teaching instrument, surveys with missing instrument values were excluded. Item values and mean scores were assessed for normality by examining skewness, kurtosis and histograms [28]. 

For the total sample and year-level samples, descriptive statistics were generated for demographic data and for the M-SECT total and domain scores. To support interpretation of results, mean M-SECT total and domain scores were divided by the total number of items in the scale/domain to produce a *converted mean*. In accordance with McArthur’s [22] original scale, converted means were valued from 1 to 7, with 1 representing the lowest (least) level of self-efficacy/confidence and 7 being the highest (most) level of self-efficacy/confidence [22]. While McArthur [22] did not specify ranges of self-efficacy/confidence within the scale of 1–7, a decision was made to use the term ‘low self-efficacy/confidence’ to describe scores of 1–2, ‘moderate self-efficacy/confidence’ for scores of 3–5 and ‘high self-efficacy/confidence’ for scores of 6–7.

For the total sample, a multiple linear regression analysis was conducted to determine the predictive power of demographic characteristics (age, gender, year level, prior university degree, education experience, healthcare experience and peer teaching experience) on M-SECT total scores. This analysis was used to address Research Question 1.

To determine if there were statistically significant differences in mean M-SECT total and domain scores between Year 2 and Year 3 participants (Research Question 2), we used independent samples *t*-tests. For these comparisons, Cohen’s *d* evaluated the magnitude of differences in mean scores between groups (small, medium, large effect) [29]. An analysis of covariance was used to control for covariates when comparing groups.

To determine the clinical teaching aspects that participants were most and least confident with (Research Question 3), item responses were analysed using descriptive statistics. For each item, the mean scores and frequency of responses (n and %) were calculated for Year 2 and Year 3 students. Again, the term ‘low self-efficacy/confidence’ was used to describe mean scores of 1–2, ‘moderate self-efficacy/confidence’ for mean scores of 3–5 and ‘high self-efficacy/confidence’ for mean scores of 6–7.

### Ethical approval

Prior to participant recruitment, ethical approval for this study was granted by the authors’ university Human Research Ethics Committee (HREC number 2022/373; Clinical trial number: Not applicable).

## Results

### Demographics

The survey was completed by 332 participants (response rate = 37.35%). Of the surveys, 319 included all instrument responses and were consequently analysed (completion rate = 35.88%). Most participants were female (86.8%) and almost half had a prior tertiary degree or diploma (42.0%). Although few participants had peer teaching experience (18.5%) or employment experience in an education setting (e.g., primary school or early childhood education centre; 1.9%), many participants were working or had worked in a healthcare setting (36.4%). There were more Year 2 participants (57.4%) than Year 3 participants (42.6%). Year 3 participants were older and had more peer teaching experience; however, a greater proportion of Year 2 participants were currently employed or had previously been employed in a healthcare setting. Table 1 presents participants’ demographic characteristics.

**Table 1:** Participant demographic characteristics

Characteristic	Total sample (*n* = 319)			Year 2 (*n* = 183)					Year 3 (*n* = 136)				
	M	SD	*n*	%	M	SD	*n*	%	M	SD	*n*	%	
Age	26.1	7.9			25.1	7.4			27.4	8.4			
Gender													
Female			277	86.8			164	89.6			113	83.1	
Male			39	12.2			19	10.4			20	14.7	
Other			2	0.6			0	0.0			2	1.5	
Prefer not to say			1	0.3			0	0.0			1	0.7	
Prior tertiary degree or diploma			134	42.0			74	40.4			60	44.1	
Peer teaching experience			59	18.5			22	12.0			37	27.2	
Education experience (employed)			6	1.9			3	1.6			3	2.2	
Health care experience (employed)			116	36.4			74	42.1			42	33.1	

### Factors predicting participants’ clinical teaching self-efficacy

For the total sample (*n* = 319), participants’ mean M-SECT total score (*M* = 101.97, *SD* = 23.93, Range = 29–147) and converted mean (*Converted M =* 4.9/7) indicated they had a moderate level of self-efficacy in clinical teaching. Multiple linear regression produced a significant predictive model (*F*(7,290) = 4.75, *R2* = 0.10, *p* < .001) with two of the variables, year level of study and prior peer teaching experience, predicting a small amount of the variance in M-SECT total scores (10%). The results showed that year level of study and prior peer teaching experience were significant positive predictors of M-SECT total scores (*t* = 3.15, *p* = .002, and *t* = 3.56, *p* < .001, respectively). Age (*t* = 0.03, *p* = .975), gender (*t* = 0.20, *p* = .840), prior university degree/diploma (*t* = -0.24, *p* = .812), employment experience in an education setting (*t* = 1.07, *p* = .287) and employment experience in a healthcare setting (*t* = -0.79, *p* = .431) were not significant predictors of mean M-SECT total scores.

### Comparison of year 2 and year 3 participants’ clinical teaching self-efficacy

For both the Year 2 (*n* = 183) and Year 3 (*n* = 136) samples, participants’ mean M-SECT total score and converted score indicated they had a moderate level of self-efficacy in clinical teaching. For the M-SECT domains, Year 2 and Year 3 participants’ mean and converted mean domain scores indicated that both participant groups had moderate confidence to “customise their teaching to a learner’s needs” (Domain 1), to “teach with prowess” (Domain 2) and to “impact a learner’s development” (Domain 3). For both Year 2 and Year 3 participants, their confidence to “teach with prowess” (Domain 2) was highest and their confidence to “impact a learner’s development” (Domain 3) was lowest of the three domains. Results are presented in Table 2.

**Table 2:** Comparison of year 2 and year 3 participants’ mean total and domain M-SECT scores

	*n*	M	SD	Range	Converted M (Scale 1–7)	M difference	95% CI	t	df	*p*	Cohen’s d
**M-SECT Total (out of 147.0)**											
Year 2	183	97.2	23.6	29–147	4.6						
Year 3	136	108.4	22.9	46–147	5.2	11.1	5.9, 16.3	4.21	317	< 0.001	0.48
**Domain 1 (out of 49.0)**											
Year 2	183	32.3	8.2	8–49	4.6						
Year 3	136	35.9	7.9	13–49	5.1	3.6	1.8, 5.4	3.95	317	< 0.001	0.45
**Domain 2 (out of 56.0)**											
Year 2	183	38.2	9.4	11–56	4.8						
Year 3	136	42.4	8.6	21–56	5.3	4.2	2.2, 6.3	4.12	317	< 0.001	0.47
**Domain 3 (out of 42.0)**											
Year 2	183	26.8	7.4	6–42	4.5						
Year 3	136	30.0	7.3	9–42	5.0	3.3	1.6, 4.9	3.92	317	< 0.001	0.44

Key: M-SECT = Modified Self-Efficacy in Clinical Teaching; Domain 1 = Customising Teaching to a Learner’s Needs; Domain 2 = Teaching Prowess; Domain 3 = Impact on a Learner’s Development

Comparing year level samples, Year 3 participants’ mean M-SECT total score was significantly higher than that of Year 2 participants (*p* < .001). The magnitude of this difference was small [29]. After adjusting for prior peer teaching experience (a positive predictor of M-SECT total scores), Year 3 participants’ mean M-SECT total score was still significantly higher than that of Year 2 participants (*F*(1,315) = 11.48, *p* < .001).

In relation to the M-SECT domains, Year 3 participants’ confidence to “customise their teaching to a learner’s needs” (Domain 1; *p* < .001), to “teach with prowess” (Domain 2; *p* < .001) and to “impact a learner’s development” (Domain 3; *p* < .001) were all significantly higher than those of the Year 2 participants. For all domains, the magnitude of differences was small [29]. Results are presented in Table 2.

### Participants’ confidence with aspects of clinical teaching

Across all domains and items, mean scores for both Year 2 and Year 3 participants indicated that they were most confident that they could show concern for a learner’s wellbeing (Item 15). Year 2 participants were least confident that they could design teaching plans (Item 17) and Year 3 participants were least confident they could handle difficult questions and situations (Item 12).

#### Item responses for domain 1: Customising teaching to a learner’s needs

The mean scores for Domain I items indicated that both Year 2 and Year 3 participants were most confident with their abilities to teach what a learner needs to know (Item 5) and to be organised and prepared for in-practice teaching (Item 7). Both year level samples were least confident in their ability to refine their teaching content and methods based on a junior student’s learning needs (Item 4).

More Year 3 participants (~ 40%) were highly confident with teaching-related tasks compared to Year 2 participants (~ 25%). Year 2 participants had lower confidence across all Domain 1 items, with approximately 7% of Year 2 students and 3% of Year 3 students rating their confidence as low. Results are presented in Table 3.

**Table 3:** Item response frequencies and means for domain 1: customising teaching to a learner’s needs

Items		Low confidence (Rating 1–2) *n* (%)	Moderate confidence (Rating 3–5) *n* (%)	High confidence (Rating 6–7) *n* (%)	Mean (out of 7)
*As of today*,* I am confident I can….*					
1. Correctly appraise the learning needs of junior nursing students (‘learners’)	Year 2	13 (7.1)	120 (65.6)	50 (27.3)	4.6
	Year 3	4 (2.9)	76 (55.9)	56 (41.2)	5.1
2. Provide appropriate instructional content, based on a learner’s learning needs	Year 2	11 (6.0)	131 (71.6)	41 (22.4)	4.5
	Year 3	3 (2.2)	80 (58.8)	53 (39.0)	5.1
3. Select appropriate teaching strategies when encountering different learner’s needs	Year 2	13 (7.1)	126 (68.9)	44 (24.0)	4.5
	Year 3	5 (3.6)	78 (57.4)	53 (39.0)	5.0
4. Refine teaching content and methods based on a learner’s learning needs and other factors	Year 2	14 (7.7)	134 (73.2)	35 (19.1)	4.4
	Year 3	4 (3.0)	79 (58.1)	53 (38.9)	5.0
5. Teach what a learner needs to know	Year 2	13 (7.1)	108 (59.0)	62 (33.9)	4.8
	Year 3	3 (2.2)	66 (48.5)	67 (49.3)	5.3
6. Be effective in my clinical teaching	Year 2	13 (7.1)	112 (61.2)	58 (31.7)	4.7
	Year 3	3 (2.2)	76 (55.9)	57 (41.9)	5.1
7. Be well organised and prepared for in-practice teaching	Year 2	13 (7.1)	105 (57.4)	65 (35.5)	4.8
	Year 3	3 (2.2)	73 (53.7)	60 (44.1)	5.3

#### Item responses for domain 2: Teaching prowess

For Domain 2 items, mean scores indicated that both Year 2 and Year 3 participants were most confident that they could show concern for a junior student’s wellbeing (Item 15). Year 2 participants were also confident that they could tailor feedback to be constructive (Item 14), while Year 3 participants were confident that they could provide clear clinical instruction (Item 8), demonstrate clinical skills (Item 9) and evaluate a junior student’s efforts (Item 10). Both sample groups were least confident that they could handle junior students’ difficult questions or situations (Item 12).

More Year 3 participants (~ 50%) were highly confident with teaching-related tasks compared to Year 2 participants (~ 33%). Year 2 participants were more likely to rate their confidence as low compared to Year 3 participants. Across all Domain 2 items, approximately 7% of Year 2 students and 2% of Year 3 students rated their confidence as low. Results are presented in Table 4.

**Table 4:** Item response frequencies and means for domain 2: teaching prowess

Items		Low confidence (Rating 1–2) *n* (%)	Moderate confidence (Rating 3–5) *n* (%)	High confidence (Rating 6–7) *n* (%)	Mean (out of 7)
*As of today*,* I am confident I can….*					
8. Provide clinical instruction in a clear manner that learners can understand	Year 2	11 (6.0)	112 (61.2)	60 (32.8)	4.8
	Year 3	0 (0.0)	66 (48.5)	70 (51.5)	5.4
9. Correctly demonstrate clinical skills to learners	Year 2	13 (7.1)	111 (60.7)	59 (32.2)	4.8
	Year 3	1 (0.7)	67 (49.3)	68 (50.0)	5.4
10. Evaluate the effectiveness of a learner’s clinical efforts through direct observation	Year 2	10 (5.5)	114 (62.3)	59 (32.2)	4.8
	Year 3	1 (0.7)	59 (43.4)	76 (55.9)	5.4
11. Teach learners to determine their professional boundaries	Year 2	11 (6.0)	121 (66.1)	51 (27.9)	4.8
	Year 3	2 (1.5)	66 (48.5)	68 (50.0)	5.3
12. Handle most learner’s difficult questions or situations	Year 2	23 (12.6)	119 (65.0)	41 (22.4)	4.3
	Year 3	9 (6.6)	82 (60.3)	45 (33.1)	4.7
13. Give clear explanations to questions around clinical scenarios	Year 2	19 (10.4)	121 (66.1)	43 (23.5)	4.4
	Year 3	3 (2.2)	75 (55.2)	58 (42.6)	5.1
14. Tailor my feedback to be constructive and developmental	Year 2	9 (4.9)	111 (60.7)	63 (34.4)	4.9
	Year 3	5 (3.7)	64 (47.1)	67 (49.3)	5.3
15. Show concern for a learner’s wellbeing	Year 2	7 (3.8)	72 (39.3)	104 (56.8)	5.5
	Year 3	0 (0.0)	43 (31.6)	93 (68.4)	5.9

#### Item responses for domain 3: Impact on a learner’s development

The mean scores for Domain 3 items indicated that both Year 2 and Year 3 participants were most confident with their abilities to change the attitudes/values of a learner (Item 16). Year 2 participants were least confident that they could design teaching plans for junior students (Item 17), while Year 3 participants were least confident that they could stimulate a junior student to learn aspects of the curriculum that do not interest them (Item 20).

For each Domain 3 item, more Year 3 participants (~ 31%) were highly confident compared to Year 2 participants (~ 18%). Year 2 participants were more likely to rate their confidence as low across Domain 3 items, with approximately 9% of Year 2 students and 4% of Year 3 students in the low confidence range. Results are presented in Table 5.

**Table 5:** Item response frequencies and means for domain 3: impact on a learner’s development

Items		Low confidence (Rating 1–2) *n* (%)	Moderate confidence (Rating 3–5) *n* (%)	High confidence (Rating 6–7) *n* (%)	Mean (out of 7)
*As of today*,* I am confident I can….*					
16. Change the attitudes/values of a learner	Year 2	14 (7.7)	108 (59.0)	61 (33.3)	4.8
	Year 3	2 (1.5)	69 (50.7)	65 (47.8)	5.3
17. Design teaching plans for learners	Year 2	21 (11.5)	132 (72.1)	30 (16.4)	4.2
	Year 3	5 (3.7)	80 (58.8)	51 (37.5)	4.9
18. Prepare learning objectives across a learner’s area of development	Year 2	16 (8.7)	137 (74.9)	30 (16.4)	4.3
	Year 3	5 (3.7)	78 (57.4)	53 (38.9)	4.9
19. Give a learner instruction on strategies and resources to support their development	Year 2	17 (9.3)	118 (64.5)	48 (26.2)	4.5
	Year 3	6 (4.4)	73 (53.7)	57 (41.9)	5.1
20. Stimulate a learner to learn areas of curriculum that don’t interest them	Year 2	14 (7.7)	134 (73.2)	35 (19.1)	4.3
	Year 3	9 (6.6)	78 (57.4)	49 (36.0)	4.8
21. Provide appropriate support to help learners learn and sustain work/life/family balance and personal wellbeing	Year 2	14 (7.7)	109 (59.5)	60 (32.8)	4.7
	Year 3	4 (2.9)	64 (47.1)	68 (50.0)	5.2

## Discussion

This study examined undergraduate nursing students’ self-efficacy in clinical teaching – an under researched topic. In this research, year level of study and prior peer teaching experiences were found to be positive predictors of nursing students’ clinical teaching self-efficacy. Nursing students in their final year of study (Year 3) had significantly higher levels of clinical teaching self-efficacy compared to Year 2 nursing students. Year-level differences were also noted for students’ clinical teaching self-efficacy domain scores and their confidence scores for teaching-related tasks. Overall, Year 2 and 3 participants were most confident they could “teach with prowess” and least confident they could “impact a learner’s development.”

Results of this study can be explained in relation to self-efficacy theory. Bandura [15] proposed that a person’s self-efficacy beliefs are strengthened through engagement with four sources of self-efficacy. In this study, final year nursing students had received more opportunities through their university program, compared to Year 2 students, to engage with *vicarious experiences* related to clinical teaching. This can be attributed to the program’s increasing hours of clinical placement experiences across year levels. Health research suggests that vicarious experiences arise during clinical placements as nursing students are exposed to qualified nurses who role model nursing-related skills and behaviours [30]. These vicarious experiences enable nursing students to identify the type of nurse they aspire to be and the type they do not [31]. They also enable nursing students to reflect on their own capabilities and to emulate and build confidence with skills and behaviours that they deem desirable [30]. In this study, Year 3 participants had additional opportunities to be taught by preceptors, clinical facilitators and/or qualified nurses during their increasingly longer clinical placements. These ‘real-life’ clinical teaching opportunities, coupled with their coursework related to teaching, likely enhanced Year 3 students’ comfort and confidence in clinical teaching. It therefore seems reasonable to claim that undergraduate nursing students’ sense of self-efficacy can be strengthened across all year levels when they are supported in identifying, observing and reflecting on the teaching practices they observe during their clinical placements.

In addition to year level, this study determined that prior peer teaching was a positive predictor of nursing students’ clinical teaching self-efficacy, accounting for a small amount of variance in students’ self-efficacy scores. This reflects Bandura’s [15] assertion that repeated *mastery experiences* enhance a person’s belief that they can succeed with a similar task in the future. Much is known about the benefits of nursing students’ repeated engagement with mastery experiences in relation to their self-efficacy beliefs [32, 33]. Al Gharibi and colleagues [32] determined that senior nursing students’ self-efficacy beliefs were enhanced by repeatedly engaging in high-fidelity simulation experiences that integrated similar clinical scenarios. Kaldheim and colleagues [33] also found that postgraduate nursing students’ self-efficacy in communication increased when they repeatedly participated in interprofessional simulations.

In relation to peer teaching, Pierce et al.^34^ found that near-peer teaching experiences, whereby a senior student teaches a junior student in classrooms, laboratories or clinical placement, strengthens senior health professional students’ self-efficacy in teaching. They attributed these self-efficacy gains, at least in part, to having repeated opportunities to hone teaching skills during the near-peer experiences [34]. In medical education, Diebolt et al [35]. found that senior students’ self-efficacy in teaching increased significantly with each successive near-peer teaching experience, reinforcing the belief that repetition leads to mastery and efficacy.

Preparation and education are essential for developing a nurse’s self-efficacy in clinical teaching [2]. The integration of some or more near-peer teaching opportunities throughout nursing curricula may support the development of graduating students’ clinical teaching self-efficacy beliefs, as they can repeatedly teach concepts and tasks to junior students. Near-peer teaching experiences have the added benefit of supporting students’ *physiological and affective states* (a source of self-efficacy), as students can practice clinical teaching in a socially safe environment with their peers rather than faculty [15, 34]. Peer teaching can also facilitate the giving and receiving of feedback among students, [36] providing opportunities for positive *verbal persuasion* (another self-efficacy source) and confidence-building [15]. 

In relation to the domains of clinical teaching self-efficacy, both the Year 2 and Year 3 nursing students in this study were *most* self-efficacious in their ability to “teach with prowess.” It is possible that the teaching-related tasks that underpin this domain had been explored by students during coursework (e.g., showing concern/empathy for others) or observed during clinical placements (e.g., providing clinical instruction and feedback). Both year-level groups were *least* confident that they could impact a junior nursing student’s development – a domain that involves assessing a junior student’s developmental needs over time and using teaching strategies that spark their interest [22]. Ascertaining the long-term learning needs of students in relation to curricula and clinical practice can be complex, even for qualified nurses [37]. Stimulating a disinterested learner also presents challenges, as it requires an understanding of active learning principles that engage and excite learners [38]. Designing curricular activities that enable nursing students to examine their own and others’ learning needs, while using active learning principles, may help to address students’ lack of confidence in these areas.

It is notable that while some final year (Year 3) nursing students in this study rated themselves as highly confident with clinical teaching tasks (30–50%), there was a large proportion of final year students (50–70%) whose confidence with clinical teaching tasks was moderate or low. This is concerning, given that many of these students will, upon graduation, be expected to educate other healthcare workers (e.g., enrolled nurses and nursing assistants) and nursing students [5]. A lack of clinical teaching confidence can translate to unpreparedness for future teaching [3] and can contribute to poor clinical teaching practices that negatively impact healthcare quality and safety [2]. More research is needed to determine the long-term implications of nursing students’ moderate to low clinical teaching self-efficacy on clinical teaching quality and patient care. A longitudinal study examining nursing students’ self-efficacy in clinical teaching as they transition to professional practice would provide valuable insights into the impact of these beliefs on patient care.

### Nursing and health professional implications

Overall, this research provides important insights into nursing students’ clinical teaching self-efficacy beliefs throughout their degree program. Such understanding may be of interest to tertiary nurse and health professional educators, who can benchmark and compare their students’ clinical teaching self-efficacy beliefs across year levels and disciplines. This research also offers insight into the curricular initiatives that may support undergraduate students’ engagement with the four self-efficacy sources [15] and the development of their clinical teaching confidence and competence during their degree program. Initiatives such as near-peer teaching can be integrated within and across health professional disciplines, offering an interdisciplinary means of enhancing health professional students’ self-efficacy beliefs in clinical teaching. Further research is needed to determine other educational initiatives and predictors that may account for variations in students’ self-efficacy in clinical teaching.

### Limitations and recommendations

This research has limitations. While the research is relevant to educators and researchers in any country where clinical teaching is a professional nursing responsibility, the recruitment of participants from a single university and country may limit the generalisability of results to other populations. Excluding Year 1 students also limited understanding of nursing students’ self-efficacy beliefs at the start of a degree program. Furthermore, self-report survey-based studies can be one-dimensional and result in social desirability response bias [39]. Future research might benefit from the integration of a qualitative component, such as interviews, and inclusion of Year 1 participants to gain deeper insights into students’ clinical teaching self-efficacy. This would also enable exploration of additional factors that may predict/influence nursing students’ clinical teaching self-efficacy and would support triangulation of quantitative results.

## Conclusion

Clinical teaching is a professional requirement of nurses. To enhance nurses’ clinical teaching competence and confidence, undergraduate nursing students must develop their self-efficacy beliefs. The value of this study is that it presents results from a cross-sectional survey that examined undergraduate nursing students’ clinical teaching self-efficacy beliefs throughout their degree program, a topic underreported in the literature. This research also provides nursing and health professional educators with suggestions for curricular initiatives that may support the development of students’ clinical teaching self-efficacy and potentially enhance their clinical teaching capabilities into the future.

## Data Availability

The datasets used and/or analysed during the current study are available from the corresponding author on reasonable request.

## Abbreviations

M-SECT: Modified Self-efficacy in Clinical Teaching
